# 

**Published:** 1979

**Authors:** 


					CHIRURGICAL
JANUARY/APRIL 1979
VOLUME 94 (i/ii)
NUMBER 349/350

				

